# Smoking and Chronic Kidney Disease in Healthy Populations

**DOI:** 10.5812/numonthly.3527

**Published:** 2012-12-15

**Authors:** Yuka Noborisaka

**Affiliations:** 1 Department of Social and Environmental Medicine, School of Medicine, Kanazawa Medical University, Ishikawa, Japan

**Keywords:** Smoking, Kidney Failure, Chronic, Proteinuria, Glomerular Filtration Rete

## Abstract

The objective of this review is to explore the link between smoking and the development of chronic kidney disease (CKD) in generally healthy populations without pre-existing renal dysfunction such as diabetic nephropathy. Twenty-eight epidemiological studies concerning the renal effects of smoking in the general population were collected from the MEDLINE database and were reviewed for indications of proteinuria and/or the decline of glomerular filtration rate (GFR), and evaluated on the level of evidence and the quality of the study. Sixteen of the 28 studies were cross-sectional in design. Most articles had some weakness in scope, such as the 6 articles which did not fully exclude DM patients from the subjects, the 4 that did not consider the effects of ex-smoking, and the 3 that focused on only a small number of subjects. From these cases, it is difficult to draw firm conclusions. However, proteinuria or microalbuminuria was persistently high in current smokers; as much as 5-8% or 8-15% respectively, which was up to 2 to 3-times the rate of lifelong non-smokers. On the other hand, only 5 studies broader in scope detected any decline of GFR in smokers, while 9 other studies suggested a higher GFR in smokers than in non-smokers. Two good quality studies showed an even a significantly lower risk of a decreased GFR in smokers. These paradoxical CKD markers in smokers, i.e., a higher appearance of proteinuria with a higher GFR, could be a focus for further studies to reveal the underlying reasons for smoking-induced CKD. Workplaces may be an excellent place to study this subject since the long-term changes in renal function of smokers can be observed by collecting data in the annual health check-ups mandated at places of employment.

## 1. Background

The adverse renal effects of smoking were first demonstrated in patients with diabetes mellitus, and successively in those with primary kidney disease such as polycystic kidney, glomerulonephritis and lupus nephritis, and those with primary hypertension (1-4). An epoch-making report published in 2000 by the PREVEND (prevention of REnal and vascular ENd stage disease) study group (5) showed that smoking was associated with an excessive urinary albumin excretion in inhabitants without diabetes in a Dutch community. This study suggested that smoking induces chronic kidney disease (CKD) as manifested by proteinuria and/or lowered glomerular filtration rate (GFR) even in generally healthy adults.

In 2007, Jones-Burton et al. (6) reviewed 17 articles concerning the association of cigarette smoking with the incidence of CKD and concluded that smoking is a significant risk factor for CKD, but that the depth of the correlation remains obscure due to the vast heterogeneity in the source populations and in the methods used to measure the outcomes. Furthermore, most of the studies included a considerable number of subjects who were diabetic. Since the renal effects of smoking are more well-known in patients with diabetic nephropathy, the impact of smoking in healthy adults without that renal dysfunction is even more difficult to determine. We tried to elucidate, therefore, the possible association between chronic smoking and CKD in generally healthy populations by reviewing previous studies conducted on this matter.

## 2. Review of the Literature

The literature concerning the renal effects of cigarette smoking in generally healthy populations was researched on the MEDLINE database using a PubMed interface on April 8, 2011. All articles published in English from 1966 onwards were searched using the following combination of terms: smoking AND (proteinuria OR albuminuria OR kidney diseases). 2,881 articles were retrieved in this manner. According to the titles, and abstracts if available, 51 original articles describing epidemiological studies conducted in the general population regarding the prevalence and incidence of the signs of CKD were selected and examined in the full length papers. Excluding duplicated articles, or those conducted mainly on diseased patients, or those not noting CKD with regards to smoking, or those not considering the effects of confounding factors, and including two recently published articles of our own (7), 28 articles were reviewed. All papers were evaluated for the level of evidence (LOE) according to the criteria proposed by the Agency for Health Care Policy and Research (AHCRP) in 1993, and for the quality of study based on items selected from the recommendations for good analytical epidemiological studies (8, 9).

The items are shown in Table 1: 1) the selection of subjects, for the definition in accordance with the goal of this review and the appropriateness of the mother population, 2) the size of the study population, 3) the duration of observation in cohort studies, 4) the definition of exposure (smoking status in this review), 5) the measurement of outcome (proteinuria and renal function), 6) statistical analyses, and 7) considerations of confounding factors. Each item was graded as “good”, “fair”, or “acceptable” where appropriate.

**Table 1:** Items and Grades for Quality Evaluation of the Analytical Epidemiological Studies on the Smoking-Induced Renal Damage in the Generally Healthy Population

		Grades	
Items	Good	Fair	Acceptable
**Selection of subjects**			
Definition^a^	Recruited from community or workplace with exclusion of preceding primary kidney diseases and DM	Recruited from community or workplace with exclusion of preceding primary kidney diseases	Recruited from community or workplace without any exclusion of preceding diseases
Representativeness	Randomized selection from or including 70% or more of the whole population	Not randomized but not arbitrary selection from the whole population	
**Size of population**	4,000 or more in men and 8,000 or more in women	400 or more in men and 800 or more in women^b^	100 or more in men and 200 or more in women
**Duration of observation^c^**	10 years or longer	5 years or longer	2 years or longer
**Definition of exposure** **(Smoking status)**	Smoker / Exsmoker Never smoker Accumulated dose	Smoker / Exsmoker Never smoker	Smoker /non-smoker
**Measurement of outcome**			
Proteinuria (Albuminuria)	Quantitative measurement	Semiquantitative (dipstick) measurement	
Renal function	Actual measurement of GFR or Ccr or Estimation of GFR by a standardized equation	Estimation of Ccr by a CockcroftGault Equation or Measurement of serum creatinine concentration	
**Statistical analysis**	Appropriate methods, Appropriate description	Appropriate methods	
**Consideration on confounding factors**^d^	Demographic factors, Anthropometrical factors, Impaired GT and high BP	Demographic factors, Anthropometrical factors	Demographic factors

Abbreviations: BP, blood pressure; Ccr, creatinine clearance CKD, chronic kidney disease; DM, diabetes mellitus; GFR, glomerular filtration rate; GT, glucose tolerance.

^a^In cohort study, the patients with the endpoint CKD should be excluded at the baseline .

^b^Minimal ample size required to detect the difference in the prevalence of CKD between smokers and nonsmokers.

^c^Required item in cohort study.

^d^Appropriate matching of case and control or applying appropriate multivariate analyses.

The study subjects should be generally healthy for this review, and thus the exclusion of subjects who may have pre-existing renal dysfunction, especially those due to primary kidney diseases or diabetes mellitus, is required for a “good” study. At least, the subjects should be recruited from communities or workplaces even though those exclusions were not fully completed as shown in “acceptable”. Appropriateness is graded as “good” when the participating subjects were randomly collected from the mother population or consisted of 70% or more of the population. Non-arbitrary collection of the subjects such as volunteers for health screenings is graded as “fair”.

Assuming the prevalence of proteinuria to be 5% in smokers and 3% in non-smokers, and the smoking rate to be 30% of male and 15% of female subjects, the minimum sample size required to detect a significant difference is estimated to be 400 in men and 800 in women, which is graded as “fair”. A sample size that is ten times larger is graded as “good”, and the sample size of 100 men and 200 women or more is graded as “acceptable”. For the range of the study (smoking in this review), the effects of past smoking should be considered, and thus the studies classifying the subjects only into current smokers or non-smokers are graded low as “acceptable”. For the measurements of proteinuria or albuminuria as the outcome, quantitative measurements of these parameters in urine are graded as “good”, and semi-quantitative measurements using a dipstick are graded as “fair”. For renal function, using a well standardized equation for estimating GFR, such as that proposed by the modification of diet in renal disease (MDRD) study group (10), or by the Japan Society of Nephrology (JSN) is graded as “good” as well as the actual measurement of GFR or creatinine clearance (Ccr). Ccr estimated by the Cockcroft and Gault (CG) equation (11) or simply serum creatinine (Cr) concentration for the index of renal function is graded as “fair” because of the lower validity for estimating real GFR values. Confounding factors should be matched or adjusted for using a multivariate analysis, and consideration of demographic factors is required for “acceptable”. As a final point ,, the articles which are graded as “good” in all the items are classified as “A”, those graded as “acceptable” in any of the items are classified as “C”, and the remaining are classified as “B”. Sixteen of the 28 articles reported the effects of smoking on proteinuria or albuminuria (7, 12-26), and 21 reported the effects on renal function (7, 12, 14, 15, 17, 21, 22, 25-38).

## 3. Proteinuria or Albuminuria in Smokers

As revealed in Table 2, 13 of the 16 studies on proteinuria or albuminuria were cross-sectional in design (LOE of 3). Only one study was classified as “A” in the quality and 9 were classified as “B” because of the use of a dipstick method for detecting proteinuria (3 articles) or albuminuria (1 article), not fully excluding DM patients from the subjects (3 articles), or not-randomized selection of the subjects (2 articles). Six studies were classified as “C”: 3 articles because of not considering ex-smoking, 2 because of the small number of subjects and one cohort study because of the short period of observation.

**Table 2:** The Effects of Smoking on Proteinuria or Albuminuria in Healthy Populations

Authors	Year	Study Design (LOE)	Main Outcomes	Quality^a^ / Comment^b^
**Metcalf PA,***et al.***(19)**	1993	Cross-sectional in 5,670 people aged 40-78 years in a community of New Zealand(III)	Mantel-Haenszel OR for slight albuminuria (29-299 mg/l in men and 30-299 mg/l in women) in current smokers vs. never smokers was 1.37 (1.01~1.88) adjusted for age, gender, ethnicity, alcohol and exercise.	B/ Not excluding DM patients
**Goetz FC,***et al.***(14)**	1997	5-yr follow up in 455 adults in a U.S. community (II^a^)	Prevalence of increased urinary albumin(≥15μg/min) was twofold more frequent in current smokers (13%) than in ex- and never smokers (7%) at the baseline measurement adjusted for age, gender, BMI and DM, but this difference was not significant.	C/ Small number of subjects
**Cirillo M,***et al.***(13)**	1998	Cross-sectional in 677 men and 890 women in an Italian community (III)	Number of cigarettes consumed per day was correlated with overnight urinary albumin excretion adjusted for age, BMI and alcohol OR for microalbuminuria (20-199μg/min) in male and female current smokers vs. non- smokers was 1.99 (0.97~4.07) and 1.91 (0.73~4.96) respectively adjusted for the confounders.	C/ Ex-smoking was not considered
**Pinto-Sietsma SJ,***et al.***(21)**	2000	Cross-sectional in 7,476 adults in a Dutch community (III)	OR for microalbuminuria (30-300 mg/24h) in current smokers consuming up to 20 cigarettes per day and in those consuming more vs. never smokers was 1.92 (1.54~2.39) and 2.15 (1.52~3.03) respectively adjusted for age, gender, BMI, BP, FPG and alcohol.	A
**Halimi JM,***et al.***(15)**	2000	Cross-sectional in 28,409 French participants in a health screening (III)	OR for proteinuria (dipstick) was 2.03 (1.43~2.93) in normotensive current smokers and 2.36 (1.14~5.32) in hypertensive smokers vs. nonsmokers adjusted for age, gender and BP.	B/ Dipstick for proteinuria
**Tozawa M,***et al.***(23)**	2002	22-yr follow-up in 3,403 male and 2,000 female Japanese participants in health screening (II^a^)	OR for incident proteinuria (dipstick) in male and female current smokers vs. nonsmokers was1.28 (0.96~1.72) and 1.30 (0.44~3.80) respectively adjusted for age, obesity, hypertension and DM.	C/ Short period of observation
**Briganti EM,***et al.***(12)**	2002	Population-based Case-control study in 11,247 adults in an Australian community(III)	OR for proteinuria (≥ 0.2 mg/mg.Cr) in current smokers was 1.58 (0.53~4.75), not significant, but it was significant, 3.64 when SBP of 131.5 mmHg or higher or, 1.76 when 2-h post-loaded glucose was 126mg/dl or above.	C/ Ex-smoking was not considered
**Yamada Y,***et al.***(24)**	2004	Cross-sectional in 11,569 male and 4,715 female Japanese workers(III)	OR for proteinuria (dipstick) was 1.11 (1.15~1.63) at each level of BI (0, 1;BI:1-199, 2;BI:200-499, 3;BI:500-799, 4;BI:800-) adjusted for age, gender, DM and BP. OR for proteinuria in smokers aged 50 years or older with a BI of 500 or above and a normal high BP was 5.44 (2.27~13.0).	B/ Dipstick for proteinuria
**Hogan SL,***et al.***(16)**	2007	Cross-sectional in 15,169 adults from U.S. communities (III)	In hypertensive subjects, OR for albuminuria (≥ 17μg/mg.Cr in men and ≥ 25μg/mg.Cr in women) was 1.85 (1.29~2.64) in current smokers vs. never smokers adjusted for age, gender. No significant effects of smoking were found in normotensive subjects.	B/ Not excluding DM patients
**Zhang L,***et al.***(26)**	2008	Cross-sectional in 13,925 adults in communities in China(III)	OR for albuminuria (≥ 17μg/mg.Cr in men and ≥25μg/mg.Cr in women) was 0.94 (0.78~1.13) in current smokers vs. nonsmokers adjusted for age, gender, obesity, DM, hyper tension and hyperlipidemia.	B/ Ex-smoking was not considered
**Ishizaka N,***et al.***(17)**	2008	Cross-sectional in 7,078 Japanese male participants in health screening (III)	OR for albuminuria (≥ 30 mg/g.Cr) in current smokers consuming 20-39 cigarettes per day and those consuming more was 1.56 (1.17~2.08) and 1.88 (0.99~3.55) respectively in comparison with never smokers adjusted for age, BP and FPG.	B/ Not excluding DM patients
**Yoon HJ,***et al.***(25)**	2009	Cross-sectional in 35,228 Korean participants in health screening (III)	OR for proteinuria (dipstick) in current smokers consuming more than 20 cigarettes per day or more vs. nonsmokers was 1.33 (1.09~1.64) in men and 1.89 (0.91~3.87) in women adjusted for age, BMI, BP, FPG.	B/ Dipstick for albuminuria
**Krol E,***et al.***(18)**	2010	Cross-sectional in 2,469 Polish adults in a community (III)	OR for albuminuria (a dipstick) in male current smokers vs. nonsmokers was 1.58 (1.07~2.33)adjusted for age, BMI, DM and hypertension, but not significant in female subjects.	B/ Not-randomized selection of the subjects
**O'Seaghdha CM,***et al.***(20)**	2010	Observational cohort-study in 1,916 inhabitants in a U.S. community(II^a^)	OR for albuminuria (≥ 17μg/mg.Cr in men and ≥ 25μg/mg.Cr in women) in current smokers vs. nonsmokers was 2.09 (1.36~3.22) adjusted for age, gender, DM and baseline urinary albumin.	b/ Not-randomized selection of the the subjects
**Sauriasari R,***et al.***(22)**	2010	Cross-sectional in 290 male and 359 female Japanese participants in health screening (III)	OR for proteinuria (≥ 49.4 mg/g.Cr) in smokers consuming 20 pack-years or more vs. never smokers was 1.56 (0.79~3.09) adjusted for age, gender, BMI and BP.	C/ Small number of subjects
**Noborisaka Y,***et al.***(7)**	2011	Cross-sectional in 990 middle-aged Japanese men from a chemical plant (III)	OR for proteinuria (a dipstick) in men who have a BI of 400-599 and those have a BI of 600 or higher was 2.94(1.01~8.55) and 3.61(1.29~10.1), respectively, adjusted for age, BMI, high BP, high FPG and high serum lipids.	B/ Dipstick for proteinuria

Abbreviations: BI, Brinkman Index, BMI, body mass index, BP, blood pressure; Cr, creatinine; DM, diabetes mellitus;.FPG, fasting plasma glucose; LOE, level of evidence defined by AHCPR (1993); OR, odds ratio

^a^Quality: For the definition, refer to text and Table 1.

^b^Comment: The main reason for grading the article as B or C.

Therefore, firm conclusions cannot to be drawn from those studies, but the following has been observed: 1) proteinuria or microalbuminuria was found in 5-8% (7, 23, 24) or 8-15% (14, 17, 21, 25) respectively, of current smokers being generally higher than in non-smokers, with up to 2 to 3-times higher than the rate of lifelong non-smokers, and 2) smoking-induced proteinuria is detectable even in middle-aged persons from the working populations (7, 17, 22, 24, 25), but 3) it is found more frequently in those with a higher BP (12, 24), higher blood glucose (12) and a higher age of 50 years or older (24).

## 4. Renal Function in Smokers

As shown in Table 3, 10 of the 21 studies on renal function are follow-up designs (LOE of 2). Three studies were classified as “A” for the quality and 9 were classified as “B” because DM patients were not fully excluded (5 articles) or outcomes measured by the Ccr by the CG equation or serum Cr concentration (4 articles). Including 6 follow-up studies, 9 articles were classified as “C”: 3 articles because of the small number of the subjects, 3 because of not considering ex-smoking, 2 because of not excluding mild CKD patients in the follow-up studies in which renal failure was set as the endpoint, and 1 because of short period of the observation.

**Table 3:** The Effects of Smoking on Renal Function in Healthy Populations [Table-fn fn796]

Authors	Year	Study Design (LOE)	Main Outcomes	Quality^a^ / Comment^b^
**Goetz FC, ***et al.***(14)**	1997	5-yr follow up in 455 adults in a U.S. community (II;^a^)	Mean Ccr was significantly higher in current smokers than in ex- and never smokers adjusted for age, gender, BMI, BP and DM. Decline of Ccr during 5 years was significantly greater in current and ex-smokers than in never smokers adjusted for the confounders.	c/ Small number of subjects
**Pinto-Sietsma SJ,***et al.***(21)**	2000	Cross-sectional in 7,476 adults in a Dutch community (III)	OR for elevated eGFR (>Mean+2SD) in current smokers consuming up to 20 cigarettes per day and in those consuming more vs. never smokers was 1.82 (1.31~2.53) and 1.84 (1.12~3.02) respectively adjusted for age, gender, BMI, BP, PG and alcohol. OR for decreased eGFR (<Mean-2SD) in current smokers consuming up to 20 cigarettes per day and in those consuming more vs. never smokers was 1.53 (1.04~2.24) and 1.83 (1.05~3.20) respectively adjusted for the confounders.	A
**Halimi JM,***et al.***(15)**	2000	Cross-sectional in 28,409 French participants in health screening (III)	Mean Ccr estimated by CG formula in current smokers was significantly higher than those in former and never smokers adjusted for age, gender and BMI. No difference in the age-related decline of Ccr among current, former and never smokers.	B/ Ccr was estimated by CG formula
**Bleyer AJ,***et al.***(28)**	2000	3-yr follow up in 4,142 inhabitants in a U.S. community aged 65 years or older (II^b^)	OR for increase in serum Cr concentration (≧ 0.3 mg/dl) in current smoker vs. never smokers was 2.10 (1.4~3.1) adjusted for age, gender and body weight.	C/ Short period of observation
**Briganti EM,***et al.***(12)**	2002	Population-based Case-control study in 11,247 adults in a Australian community (III)	OR for low eGFR (<60 ml/min/1.73 m2) estimated by CG formula in male current smokers was Ex-smoking was 3.59 (1.27~10.09), but 0.90 (0.39~2.06) in female not considered smokers in comparison with nonsmokers adjusted for age, BMI, BP and FPG.	C
**Haroun MK,***et al.***(33)**	2003	20-yr follow up in 23,534 men and women in a U.S. community (II^a^)	HR for incident ESRD or death due to kidney disease in male and female current smokers vs. nonsmokers was 2.4 (1.5~4.0) and 2.9 (1.7~5.0) respectively adjusted for age, DM and BP.	C/ Not excluding mild CKD patients at the baseline
**Fox CS,***et al.***(30)**	2004	18.5-yr follow up in 1,223 men and 1,362 women in a U.S. community (II^a^)	OR for incident low eGFR(≦ 59.25 ml/min/1.73 m2 in women and ≦ 64.25 ml/min/1.73 m2in men) was 1.42 (1.06~1.91) in current smokers vs. nonsmokers adjusted for age, gender, BMI, DM and hypertension	C/ Ex-smoking was not considered
**Ejerblad E,***et al.***(29)**	2004	Population-based case-control study (926 CRF cases) in a Swedish community (III)	OR for CRF (serum Cr level ≧ 3.4 mg/dl in men and≧2.8 mg/dl in women) in smokers consuming 16-30 pack-years of cigarettes and in those consuming more vs. never smokers was 1.32 (1.00~1.75) and 1.52 (1.08~2.14) respectively adjusted for age, gender, alcohol, education and the use of analgesics.	B/ Renal function was evaluated only by serum Cr level
**Baggio B,***et al.***(27)**	2005	3.6-yr follow up in 1,283 men and 1,147 women in a Italian community aged 65-84 years (II^a^)	OR for increase in serum Cr concentration (≧26.5μmole/l)was 2.29 (1.00~5.27) in current smokers consuming 20 cigarettes per day or more vs.never smokers adjusted for age, DM, hypertension and high plasma fibrinogen.	B/ Renal function was evaluated only by serum Cr level
**Hallan S,***et al.***(31)**	2006	Cross-sectional in 30,485 males and 34,708 females 34,708 females in a Norwegian community (III)	OR for CKD (eGFR<45 ml/min/1.73 m2) in smokers consuming 25-49 pack-years of cigarettes and in those consuming more was 1.42(1.00~2.00) and 2.05 (1.08~3.89) respectively adjusted for age and gender.	B/ Not excluding DM patients
**Shankar A,***et al.***(37)**	2006	5-yr follow up in 3,392 inhabitants aged 43-84 years in a U.S. community (II^a^)	Cross-sectional phase: OR for CKD (eGFR<60 ml/min/1.73 m2) in smokers consuming 15-34 pack-years of cigarettes and in those consuming more was 2.57 (1.79~3.70) and 2.93 (2.08~4.12) respectively adjusted for age, gender, BMI, education, DM and hypertension. Follow up phase: OR for incident CKD in current smokers vs. never smokers was 1.97 (1.15~3.36) adjusted for the confounders.	B/ Not excluding DM patients
**Yamagata K,***et al.***(38)**	2007	10-yr follow up in 41,012 men and 82,752 women aged 40 years or older in a Japanese community (II^a^)	OR for incidence of stage 1 and 2 CKD in current smokers was 1.26 (1.14~1.41) in men and 1.40(1.16~1.69) in women adjusted for age, obesity,DM, hypertension, hyperlipidemia and alcohol. OR for incidence of stage 3 CKD in current smokers was 1.13 (1.05~1.22) in men and 1.16 (1.06~1.26) in women adjusted for the confounders.	B/ Not excluding DM patients
**Noborisaka Y,***et al.***(36)**	2007	Cross-sectional in 2,133 male Japanese workers (III)	Mean Ccr estimated by CG formula was significantly higher in current smokers than in former and never smokers adjusted for age and BMI.	C/ Ccr was estimated by CG formula
**Zhang L,***et al.***(26)**	2008	Cross-sectional in 13,925 adults in communities in China (III)	OR for low eGFR (<60 ml/min/1.73 m2) was 1.15 (0.79~1.68) in current smokers vs. non-smokers adjusted for age, gender, obesity, DM, hypertension and hyperlipidemia.	B/ Ex-smoking was not considered
**Kronborg J,***et al.***(34)**	2008	7-yr follow up in 2,249 men and 2,192 women in a Norwegian community (II^a^)	Current female smokers showed a signifcantly larger increase in eGFR during 7 years as compared with never smokers adjusted for age, WC,BP, DM, alcohol and physical activity, while male smokers did not.	B/ Not excluding DM patients
**Ishizaka N,***et al.***(17)**	2008	Cross-sectional in 7,078 Japanese male participants in health screening (III)	OR for low eGFR (<60 ml/min/1.73 m2) in current smokers consuming 20-39 cigarettes per day and those consuming more was 0.63 (0.49~0.83) and 0.32 (0.13~0.79) respectively adjusted for age, SBP and FPG. OR for high eGFR (> 90.73 ml/min/1.73 m2)	B/ Not excluding DM patients
**Yoon HJ,***et al.***(25)**	2009	Cross-sectional in 35,228 Korean participants in a health screening program (III)	Mean eGFR was significantly higher in current smokers than in former and never smokers. In the subjects showing a low eGFR (<50 ml/min/1.73 m2), current smokers showed lower eGFR than former and never smokers. OR for incident low eGFR (<60 ml/min/1.73 m2) in current smokers consuming up to 20 cigarettes and in those consuming more was 0.76 (0.62~0.94) and 0.73 (0.60~0.90) respectively adjusted for age, BMI, BP and FPG.	A
**Sauriasari R,***et al.***(22)**	2010	Cross-sectional in 290 male and 359 female Japanese participants in health screening (III^a^)	OR for high eGFR (≧96.7 ml/min/1.73 m2) in smokers consuming less than 20 pack-years and in those consuming more vs. never smokers was 1.08 (0.59~1.98) and 2.38 (1.15~4.93) respectively adjusted for age, gender, BMI and BP.	C/ Small number of subjects
**Miyatake N,***et al.***(35)**	2010	5-yr follow up in 286 male Japanese workers (II^a^)	Reduction of eGFR during 5 years was signifcantly smaller in 145 current smokers than in 141 nonsmokers.	C/ Small number of subjects
**Hallan SI, Orth SR(32)**	2011	10-yr (median) follow up in 65,589 adults from a community in Norway (II^a^)	HR for the incidence of stage 5 CKD in former and current male smokers was 3.74(1.05~13.2) and 5.75(1.46~22.6), respectively, as compared to never-smokers. HR was 3.19(0.76~13.5) and 2.77(0.64~11.9), respectively, in females. Cessation of smoking significantly reduced the incidence of stage 5 CKD dependently to the lapsed years from the cessation.	C/ Not excluding mild CKD patients at the baseline
**Noborisaka Y***, et al.***(7)**	2011	Cross-sectional in 990 middle-aged Japanese men from a chemical plant (III)	Mean eGFR was significantly higher in current smokers than in former and never smokers. Normal but high eGFR (≧110 ml/min/1.73 m2) was 6.7% in current heavy smokers and subnormal eGFR (< 60 ml/min/1.73 m2) was 5.7% in those with a BI of 600 or higher while both were 3.0% or less in never-smokers, although the differences between smokers and non-smokers were not significant.	A

Abbreviations: BI, Brinkman Index; BP, blood pressure; BMI, body mass index; Ccr, creatinine clearance; CG formula, Cockcroft and Gault formula; Cr, creatinine; DM, diabetes mellitus; ESRD, end stage renal disease; FPG, fasting plasma glucose; HR: hazard ratio, LOE, level of evidence defined by AHCPR (1993), OR: odds ratio

^a^Quality: For the definition, refer to text and Table 1.

^b^Comment: The main reason for grading the article as B or C.

The overall outcomes in the studies on renal function are conflicting. Only 5 studies with grade A or B in quality (27, 29, 31, 37, 38) detected a significant effect of smoking on the decline of renal function, which was also suggested in other 6 studies with grade C (12, 26, 28, 30, 32, 33). These studies were all conducted in community populations, some of those included many elderly persons (27, 28, 37, 38) or CKD patients (32, 33). Yamagata et al. (38) followed 124,000 inhabitants aged 40 years or older in a community in Japan for 10 years, excluding all those showing CKD signs beforehand, and observed that smoking caused a significant but only 10% increase in the risk for a declining GFR to the level of less than 60 ml/min/1.73m. On the other hand, 9 studies with any grade in quality even showed a higher GFR or Ccr in smokers than in non-smokers, especially those conducted in working populations (7, 17, 22, 25, 35, 36). A significantly lower risk of a low GFR was even observed in current smokers (17, 25). No difference was observed in age-related decline of GFR or Ccr among current and former smokers, and life-time non-smokers (7, 15). One study showed even a more modest decline of GFR in smokers than in non-smokers during a 5-year period (35).

In addition to the generally low LOE and quality of the literature, this article has some other limitations. The literature was collected only from the MEDLINE database, and some important articles may thus have been over-looked. The methods and manner of quality evaluation of the articles in this review have not been approved by experts other than us, which might have added some arbitrariness to the evaluation. But, in these specific circumstances, this review reveals some peculiar paradoxical findings of CKD signs in smokers in healthy populations, i.e., a persistently high appearance of proteinuria often accompanied with an elevated GFR.

## 5. Discussion

### 5.1. Significance of the Paradoxical CKD Signs in Smokers

Yoon et al. (25) in Korea has already pointed out the paradoxical CKD signs in a cross-sectional observation in 35,288 participants of a health screening program, and named “the different effect of smoking on GFR and proteinuria in a healthy population”. They mentioned that the association of smoking status with GFR was different between those showing a GFR of 50 ml/min/1.73m^2^ or above and those with a lower GFR. In those with the relatively high GFR, smokers showed a higher mean GFR than non-smokers, but an inverse association was observed in those with the low GFR, i.e., smokers have a lower GFR than non-smokers. From these findings and the limited appearance of proteinuria even in smokers, Yoon et al. proposed a hypothesis that most smokers from the general population do not show deteriorations of renal function even though they showed an elevated GFR, while only a small, especially susceptible subset of the population would show a lowered GFR and proteinuria. However, this hypothesis has not yet been confirmed.

Possible factors underlying the development of CKD in smokers were extensively discussed by Orth and Hallan (2) such as hypoxia, heavy metals in tobacco smoke, intrarenal vasoconstriction, oxidative stress and inflammatory process. Although the exact variable has remained uncertain, the intraglomerular hypertension caused by the intrarenal hemodynamic changes due to nicotine in cigarette smoke may be the most plausible reason. The high GFR in smokers may thus be a reflection of glomerular hyperfiltration following the intraglomerular hypertension and the early sign of renal damage like that observed in the early stage of diabetic nephropathy (39). If so, the high GFR in smokers may eventually decrease to a low level with continued smoking and cause proteinuria. However, this has not been confirmed either since longitudinal observations on GFR have so far failed to identify a more marked decline of GFR in smokers than in non-smokers (34, 35).

### 5.2. Advantages of Studies in Workplaces

The number of CKD patients is estimated to be 13 million or more in Japan, which is more than 10% of the national population. However, it has not been well recognized among healthcare experts, especially those engaged in preventing activities for life-style diseases at worksites. The population of Japan is aging rapidly and so is the workforce, and most workplaces are predicted to have as many as 30% or more workers aged 60 years or older in 2050. Since the renal toxic effects of smoking are more predominant in elderly persons, healthcare experts at worksites may face the far-ranging and profound impact of smoking-induced CKD in the near future.

The aging of the working population, on the other hand, may provide healthcare experts at worksites with a greater chance of observing the long-term annual changes in renal function in smokers throughout middle-age to the age of 70 years or longer. Therefore, workplaces may have special merits of revealing a more conspicuous decline of GFR in smokers once showing a higher GFR as compared to non-smokers from the data collected by the annual health check-ups mandated by the workplace. Healthcare experts at workplaces should pay more attention to smoking-induced CKD.
